# Calculating small-angle scattering intensity functions from electron-microscopy images†

**DOI:** 10.1039/d2ra00685e

**Published:** 2022-06-06

**Authors:** Batuhan Yildirim, Adam Washington, James Doutch, Jacqueline M. Cole

**Affiliations:** Cavendish Laboratory, Department of Physics, University of Cambridge J. J. Thomson Avenue Cambridge CB3 0HE UK jmc61@cam.ac.uk; ISIS Neutron and Muon Source, STFC Rutherford Appleton Laboratory Didcot Oxfordshire OX11 0QX UK; Research Complex at Harwell, Rutherford Appleton Laboratory Didcot Oxfordshire OX11 0FA UK

## Abstract

We outline procedures to calculate small-angle scattering (SAS) intensity functions from 2-dimensional electron-microscopy (EM) images. Two types of scattering systems were considered: (a) the sample is a set of particles confined to a plane; or (b) the sample is modelled as parallel, infinitely long cylinders that extend into the image plane. In each case, an EM image is segmented into particle instances and the background, whereby coordinates and morphological parameters are computed and used to calculate the constituents of the SAS-intensity function. We compare our results with experimental SAS data, discuss limitations, both general and case specific, and outline some applications of this method which could potentially complement experimental SAS.

## Abstract 2

We outline procedures to calculate small-angle scattering (SAS) intensity functions from 2-dimensional electron-microscopy (EM) images for two types of scattering systems.

## Graphical abstract

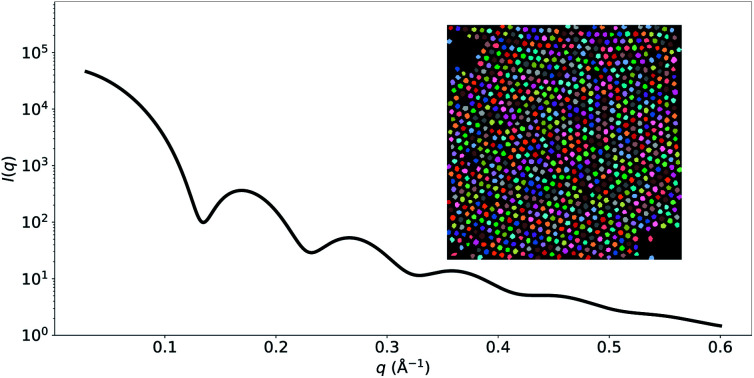

The structures of nanoparticulate systems are commonly characterized by various forms of electron microscopy (EM) and small-angle scattering (SAS) methods. The size and shape of nanoparticles, as well as their spatial-distribution functions, are of particular interest since they govern their structure–function relationships and thus their nanotechnological prospects.^1–6^ EM and SAS data are highly complementary. For example, the former images a specific section of a nanomaterial, while the latter realizes its bulk structure by averaging signals obtained from a larger overall area and depth reflective of the sample thickness and beam size. There exists a high degree of overlap in the length scale that is interrogated by EM and SAS data on the same nanomaterial. Yet, these data are necessarily acquired separately and they are analyzed independently. Nevertheless, if suitably processed, the data from one metrology could be used to reconstruct the other. This could draw out the maximum possible structural information about a nanomaterial, or allow data from both sources to be fused to obtain more accurate insights or even highlight processes that result in discrepancies between data from the two methods.

This work presents two case studies in which we calculate SAS data from 2-D EM images where (1) the particles being characterized exist on a plane; (2) the sample being imaged can be modelled as parallel, infinitely long cylinders that extend into the image plane. In both cases, we discuss limitations that result in discrepancies between image-obtained SAS intensities and those obtained experimentally. Despite these limitations, we discuss how this method can be complementary to small-angle scattering measurements, by informing experimental design decisions and aiding in model selection. The second case that we present was partially explored by Worthington and Inouye,^7^ and later Meek and Quantock^8^ as well as Quantock *et al.*^9^ They studied the interfibril distance of collagen fibres in animal corneas by calculating an interference function from pairwise distances of points obtained from an EM image. Their interference function is related to the structure factor which we include in our calculation of SAS intensities, along with form factors which we additionally compute from images. Grubb *et al.*^10^ studied the effect of the orientation of lamellar stack structures on SAXS patterns. The authors did this by generating synthetic images of arrays of lamellar stacks and simulating SAXS data using the 2-D Fourier transform, where they use the Fourier Slice theorem to obtain a 2-D slice of the 3-D transform.^11^ Afsari *et al.*^12^ and Kim *et al.*^13^ outline a procedure for calculating small-angle X-ray scattering (SAXS) data from cryo-EM images. Their work makes use of the fact that averaging the correlation functions of many cryo-EM images is equivalent to the Abel transform of SAXS data. Their work is complementary to ours as both methods can be applied under different circumstances. Our work is relevant in situations where image-processing and computer-vision techniques can be employed to segment single EM images and determine morphological and structural information about the scatterers; theirs is relevant when one has numerous cryo-EM images of the same sample.

## 1 Methodology

The SAS-intensity function factorizes as the product of the form factor *P*(*q*) and the structure factor *S*(*q*) of the scatterers,

1 *I*(*q*) ∝ *P*(*q*)*S*(*q*).

The form factor quantifies the morphology (size and shape) of the scatterers in the system, while the structure factor encodes information about their structural arrangement. When *I*(*q*) is measured experimentally, it is also scaled by a term that includes the volume fraction of the particles and the contrast against the background media of buffer in which they are dispersed; an additive background constant is also included to subtract the effects of non-sample scattering due to a number of experimental factors such as sample holders, windows and noise. We omit the contrast and background terms in eqn (1) and in our calculations as we obtain *I*(*q*) solely from images.

To estimate both *P*(*q*) and *S*(*q*) from EM images, we segment an image using the particle-instance-segmentation module^14^ of ImageDataExtractor.^15^ The result is a set of particle-instance-segmentation maps which feature a binary image for each particle, separating it from other particles and the background. Using the pixel-wise information of each particle, we are able to obtain its size and shape attributes from which we calculate *P*(*q*), as well as its particle coordinates to calculate a radial-distribution function, from which *S*(*q*) follows directly. Although the initial size and position information within an image is in units of pixels, the automatic scalebar detection and measurement functionality of ImageDataExtractor can be used to convert these values into the relevant units (*i.e.*, Ångstroms).

In calculating *P*(*q*), our method takes advantage of the fact that many form factors have analytical solutions and can be calculated if certain morphological parameters of a particle are known. We compute these parameters from particle segmentations where possible, and use the relevant form-factor expressions to calculate *P*(*q*). Analytical form-factor expressions for several general particle shapes are defined by Guinier and Fournet,^16^ and Pederson.^17^ Additionally, the SasView package documentation (https://www.sasview.org/documentation/) provides a comprehensive list of form-factor expressions for various particle shapes and sample morphologies. We note that inferring the morphological parameters of 3-D particles from 2-D images may not be achievable in some cases; hence this method of calculating form factor parameters from image segmentations may not always be possible.

The structure factor *S*(*q*) is the Fourier transform of the radial-distribution function, *g*(*r*), which characterizes the structure of a system of particles in real space.^18^ If we select an arbitrary particle as the origin, *g*(*r*) describes the number of particles that we would observe relative to the bulk density of the system, as a function of distance from the origin particle. This is calculated over every particle in the structure being considered and averaged,2
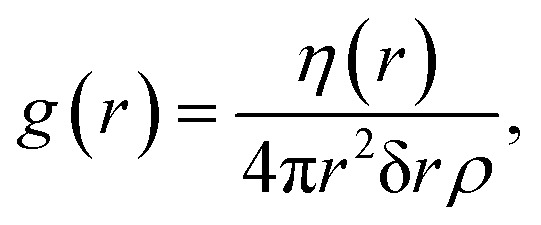
where *η*(*r*) is the average number of particles between distances *r* and *r* + δ*r*, and 
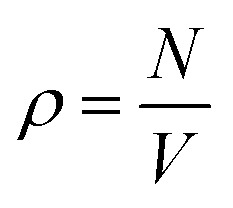
 is the number density (number of particles, *N*, divided by volume, *V*). If a system of scatterers is sufficiently dilute *i.e.*, there is no correlation between the locations of the scatterers, the structure factor term does not contribute significantly to *I*(*q*); thus *S*(*q*) can be omitted from eqn (1) because its shape will be determined predominantly by the form factor. To calculate *g*(*r*), we first obtain the coordinates of each particle from the centre of mass of their segmentation maps. These coordinates are used to compute *η*(*r*) in eqn (2) which is normalized to obtain *g*(*r*). In this work, we use rdfpy,^19^ an open-source Python module, to compute *g*(*r*) in the case studies presented in the following section. The structure factor is then obtained by taking the Fourier transform of *g*(*r*), however, the expressions differ in the two cases considered in this work, so the relevant expressions are provided in the following subsections of each specific case.

In summary, we compute *P*(*q*) and *S*(*q*) from pixel-wise segmentation information obtained from EM images where possible, and multiply the results to obtain a SAS-intensity function, *I*(*q*) (eqn (1)). In the following section, we outline further details about the processes for computing *P*(*q*) and *S*(*q*) in two case studies, although the high-level procedure remains the same each case. Limitations, both general and case specific, are also highlighted and discussed in each case.

## 2 Results and discussion

### 2.1 Particles on a plane

We first consider the case where the 3-dimensional particles being characterized by SAS exist on a plane, and hence their *z*-coordinate is arbitrary and the same for each particle. For this case, our data source is a TEM image of palladium nanoparticles by Wu *et al.*^20^ The authors studied the growth of these nanoparticles by small-angle X-ray scattering (SAXS) and complementary TEM, resulting in an image plus corresponding SAXS-intensity function pair (extended data Fig. 3 of their work^20^). We used this TEM image to compute our image-obtained SAS profile, *I*_img_(*q*) and compared it to the SAXS model-fit *I*_exp_(*q*) of Wu *et al*.^20^

Since the nanoparticles in the image of this case study are spheres, we employed the spherical form factor,3
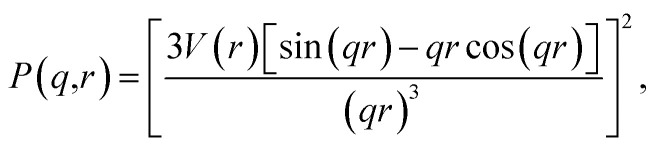
where *V* is the volume of the nanoparticle and *r* is the nanoparticle radius, which is computed from the area, *A*, of each particle's segmentation as 
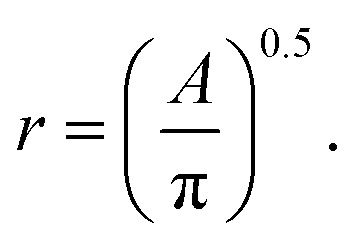
 We accounted for polydispersity and integrated over the probability distribution of particle sizes. We determined the mean and standard deviation of particle sizes from the segmentations and selected a Gaussian to represent the size distribution. The resulting form factor expression is4
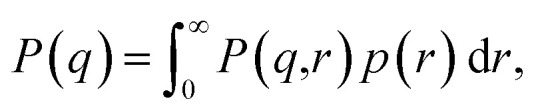
where *p*(*r*) is the probability of a particle having radius *r* under the size distribution.

To determine the structure factor, we first calculated *g*(*r*) using eqn (2) and smoothed the result using a Savitzky–Golay moving average filter.^21^ We take advantage of the fact that the radial-distribution function is a spherically symmetric function (*g*(**r**) = *g*(*r*)) to convert the 3-D Fourier transform expression into a more readily computable form. By choosing spherical coordinates in which the independent variable of the forward 3-D Fourier transform, the momentum transfer vector, **q**, lies along the polar axis (*θ* = 0), the expression simplifies and we can obtain *S*(*q*) by5
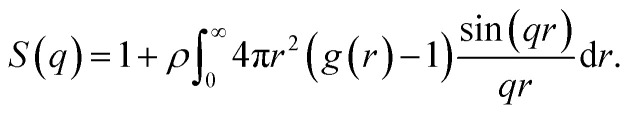


**Fig. 1:**
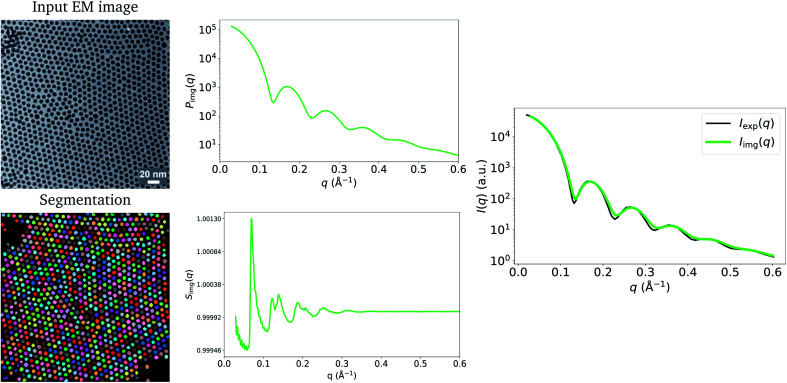
Input EM image from Wu *et al.*^20^ (reprinted by permission from Springer Nature: ref. 20, Wu *et al.*; copyright 2017) and the resulting particle segmentations obtained using ImageDataExtractor.^15^ The particle coordinates computed from the segmentations are overlaid in each case (black dots). *P*_img_(*q*) and *S*_img_(*q*), which are obtained from the segmentations, are shown and the resulting *I*_img_(*q*) = *S*_img_(*q*)*P*_img_(*q*) is compared to *I*_exp_(*q*). Notice that *S*_img_(*q*) is close to 1.0 at all values of *q*, which results in a negligible contribution of *S*_img_(*q*) to *I*_img_(*q*); hence, the absence of the structure-factor peaks in the resulting image-obtained SAS-intensity function.

Fig. 1 shows the input image and the resulting image-obtained *P*_img_(*q*), *S*_img_(*q*) and *I*_img_(*q*), as well as the experimental model fit, *I*_exp_(*q*), by Wu *et al.*^20^ The figure shows that there is good agreement between *I*_img_(*q*) and *I*_exp_(*q*). An observation that becomes immediately apparent is that despite there being significant structure in the arrangement of the Pd nanoparticles in the TEM image, and that pronounced peaks are present in *S*_img_(*q*), the structure-factor contribution does not appear to be present in *I*_img_(*q*) or in *I*_exp_(*q*). A potential explanation for this is that *S*(*q*) = 1, *∀q* due to the nanoparticles being in solution. However, in *Extended Fig. 3* of their work, Wu *et al.*^20^ state that both their TEM image and 1-D SAXS data were obtained with the nanoparticles in their final state. Thus, the lack of structure factor in our results can be explained by the low number density, *ρ*, which is a result of the particles sitting on a plane in 3-D space. Relative to the volume of 3-D space in which they are characterized, the number of particles is small, leading to a small *ρ*, which results in a structure-factor that is close to 1 at every value of *q*. This explains why the structure factor peaks are not visible in the experimentally obtained *I*_exp_(*q*), and since we take into account this 3-D volume in our procedure, they do not appear in *I*_img_(*q*) either. Note that we compare our result to the model fit *I*(*q*) of Wu *et al.*^20^ We refer the reader to the work of Wu *et al.*^20^ for evidence that the structure-factor peaks also do not appear in the raw 1-D SAXS data obtained by the authors (extended data Fig. 3 of their work^20^). These results suggest that when characterizing a surface or single plane of particles in 3-dimensions using SAS, the structure-factor contribution is unlikely to be observed in the resulting *I*(*q*). Additionally, due to the small values of *S*(*q*) in this case, and to potential noise in the experimental process, it may not be possible to obtain the structure factor experimentally by dividing *I*(*q*) by *P*(*q*).

Although the example in Fig. 1 shows a good agreement between *I*_img_(*q*) and *I*_exp_(*q*), it is possible that slight discrepancies between the two may result owing to differences in particle-size measurements. In many reports, including that by Wu *et al.*,^20^ it is common to see a discrepancy in the size distributions estimated from EM and those estimated by SAS. Apparent differences of particle sizes in EM images can occur due to a number of reasons. Kuerbanjiang showed that particles imaged by secondary electron detectors can appear twice as large as those imaged by in-lens detectors on the same sample area, due to secondary electrons being emitted at all angles from the particles in the former, while the latter detects electrons that are emitted mostly normal to the sample surface.^22^ Results from Mahl *et al.* showed similar discrepancies in size distributions obtained by SEM and TEM on silver and gold nanoparticles.^23^ Moreover, inter-laboratory studies of particle-size distributions have shown that different laboratories can obtain different particle-size statistics on the same samples measured by TEM^24^ and atomic force microscopy.^25^ Due to this unreliability of EM methods in measuring particle sizes, size statistics determined by SAS experiments can vary from those determined by EM, resulting in minor differences in scattering-intensity functions that are obtained by employing the two techniques. Since particle morphology is included in the calculation of the form factor (*i.e.*, eqn (3)), a small deviation in measured particle size can cause *I*(*q*) to be stretched along the *q*-axis. In the experiments by Wu *et al.*,^20^ the means of the size distributions obtained by experimental SAXS and by TEM match very closely for the example in Fig. 1 of our work. We obtained an average particle radius of 33.8 (±2.3) Å from our image-based particle segmentations, while Wu *et al.* reported an average of 34.0 Å obtained by SAXS.^20^ Consequently, our *I*_img_(*q*) lines up well with their *I*_exp_(*q*), since we obtained our particle-size distribution from the TEM image. However, this may not always be the case.

**Fig. 2:**
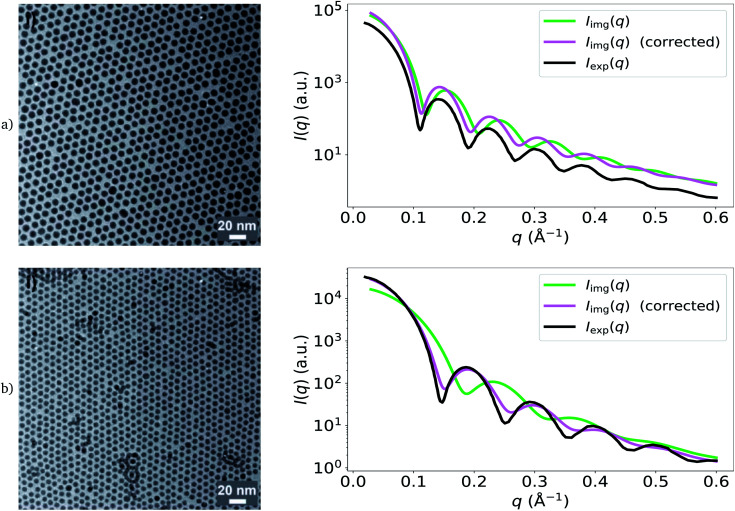
Demonstration of the effect of apparent particle-size differences (between EM and SAS) on *I*_img_(*q*) on two additional images obtained by Wu *et al.*^20^ (reprinted with permission from Springer Nature: ref. 20, Wu *et al.*; copyright 2017). The green lines are the result of using particle sizes calculated from the TEM images to calculate *I*(*q*). The pink lines are the corrected versions, which use the SAS-obtained size-distribution means obtained by Wu *et al.*^20^ Black lines are *I*_exp_(*q*), the original models fit to experimental SAXS data by Wu *et al.*^20^ Corrected versions of *I*_img_(*q*) are in better agreement with *I*_exp_(*q*).

We demonstrate the effect of differences in apparent size distributions measured by EM and SAS in Fig. 2, by computing two additional image-derived scattering intensities from two other TEM images of the Pd nanoparticles obtained by Wu *et al.*^20^ Three types of nanoparticles were actually synthesized by Wu *et al.*; these were prepared using distinct organic-acid ligands, which may have influenced the contrast of TEM in each case and resulted in the observed discrepancies. For the green and black *I*(*q*) profiles shown in Fig. 2, from top to bottom respectively, we obtained average particle radii of 37.4 (±2.3) Å and 24.4 (±2.3) Å from the particle segmentations (green), while Wu *et al.* reported averages of 40.1 Å and 30.1 Å from SAXS (black).^20^ As a result, the image-obtained intensities (green) are stretched along the *q*-axis compared to the experimental SAXS model fits (black), causing a mismatch between key features of the form-factor contribution to *I*(*q*). For the purpose of demonstration, we also show the corrected versions of these functions (pink) which use the average particle size determined by Wu *et al.*^20^ using SAXS, instead of those computed from the particle segmentations of the TEM images. It is clear that the corrected versions are in better agreement with the experimental SAXS model fits.

### 2.2 Parallel cylinders

In the case where scatterers are parallel cylinders which extend into the image plane, we can calculate *I*(*q*) from an EM image by incorporating assumptions that are commonly used when such samples are modelled experimentally with SAS. For this case study, we use a TEM image plus corresponding SAXS-intensity function obtained by Kelly *et al.*, who studied the effect of different preservatives on the sizes of collagen fibrils found in animal corneas.^26^ We calculate *I*_img_(*q*) from their TEM image of a sheep cornea slice (Fig. 2G of their work^26^) and compare this with their experimentally obtained *I*_exp_(*q*) of the same sample (Fig. 1 of their work^26^).

We assume that the scatterers are cylindrical rods of infinite length and that they are perfectly parallel to each other. By making these assumptions, the scattering intensity is localized in a plane and the problem reduces to two dimensions.^27,28^ The form factor of an infinitely long cylinder is well known and is commonly used to model experimental SAS data resulting from samples with cylindrical components of large length.^8,29^ The expression is6
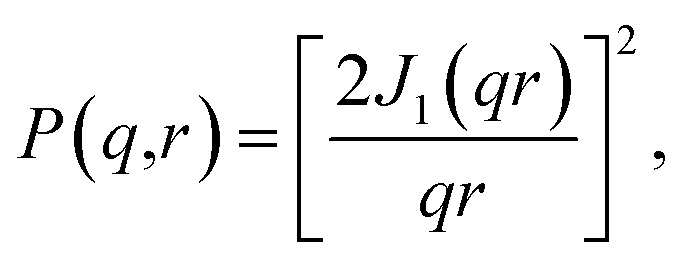
where *r* is the cylinder radius and *J*_1_ is the first-order Bessel function. Using eqn (4), we again take into account polydispersity by integrating over the distribution of cylinder radii calculated from the particle segmentations.

The structure factor can be determined by first calculating a 2-D radial-distribution function from coordinates of the fibrils obtained from the image. *g*(*r*) in 2-D differs slightly from the 3-D case (eqn (2)), in that the normalization by the thickness of each shell is 2π*r*δ*r* instead of 4π*r*^2^δ*r*, and that *η*(*r*) is calculated from 2-D coordinates instead,7
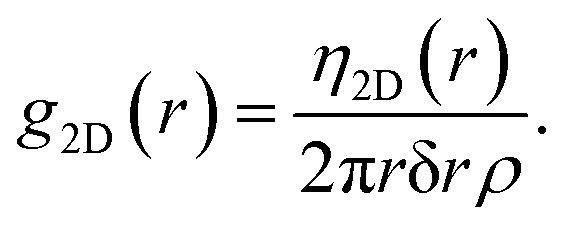


We can obtain an expression similar to eqn (5) for the 2-D Fourier transform of a cylindrically symmetric function using the fact that *g*_2D_(*r*) is independent of the angular component *θ*,8
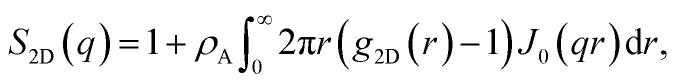
where *J*_0_ is the zeroth-order Bessel Function. The number density in this case is the 2-dimensional areal number density, 
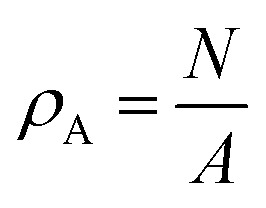
 – the number of fibrils in the image, *N*, divided by the area spanned by the image, *A*. This structure factor also applies to systems that can be modeled as disks, stacked disks, and more generally, 2-D assemblies of circles.

**Fig. 3:**
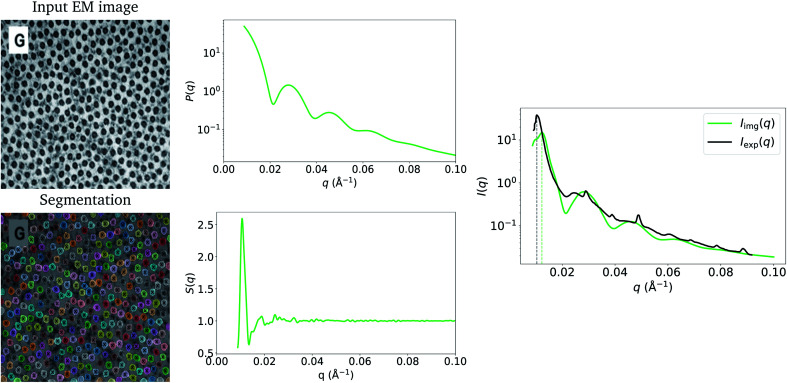
Input EM image from Kelly *et al.*^26^ (reprinted by permission from Springer Nature: ref. 26, Kelly *et al.*; copyright 2021) and the resulting particle segmentations obtained using ImageDataExtractor.^15^ The particle coordinates computed from the segmentations are overlaid in each case (black crosses). *P*_img_(*q*) and *S*_img_(*q*), which are obtained from the segmentations, are shown, and the resulting *I*_img_(*q*) = *S*_img_(*q*)*P*_img_(*q*) is compared to *I*_exp_(*q*). The structure-factor peak (at around 0.01 Å^−1^) in *I*_img_(*q*) is misaligned with *S*_exp_(*q*) owing to shrinkage of the sample used in SAXS.

Fig. 3 shows the resulting *P*_img_(*q*) and *S*_img_(*q*), and a comparison between *I*_img_(*q*) and *I*_exp_(*q*) (the experimentally obtained counterpart by Kelly *et al.*^26^). The peaks from the form-factor contribution (0.02 Å^−1^ onwards) to *I*_img_(*q*) appear at similar values of *q* to the experimental data. This can be attributed to the accurate mean fibril radius measured by ImageDataExtractor^15^ in this case, which is the main parameter used to calculate the form factor (eqn (6)). We obtained a mean fibril diameter of around 366 (±31) Å from the image, while Kelly *et al.*^26^ reported a mean fibril diameter of around 370 Å obtained by SAXS. However, due to the local nature of our method, in contrast to SAXS, which measures bulk structure, it is likely that we underestimate the standard deviation of the fibril-diameter distribution; indeed, this is apparent in *I*_img_(*q*) where, due to less polydispersity, the form factor bumps are more pronounced than in *I*_exp_(*q*).

**Fig. 4:**
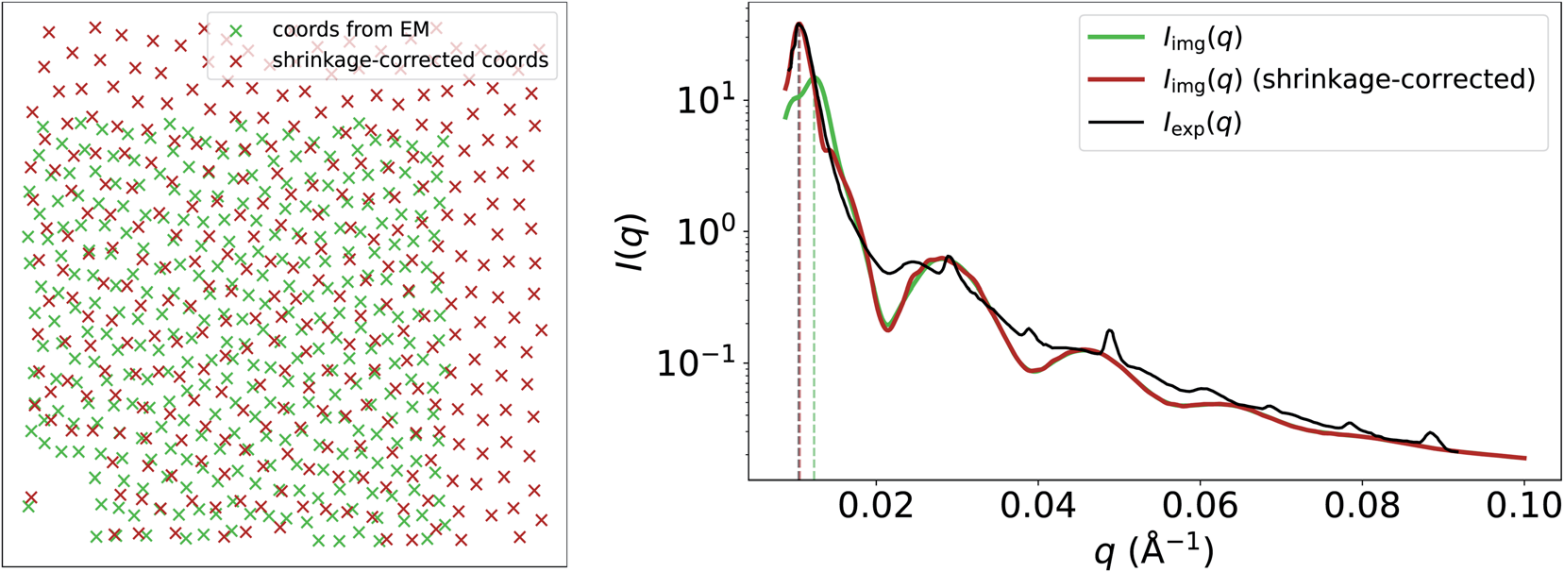
Left: relative collagen-fibril coordinates obtained from EM-image segmentation (green) and shrinkage-corrected coordinates (red). Right: resulting *I*_img_(*q*), where the *S*_img_(*q*) component was calculated using coordinates obtained from the EM image (green); and the same using shrinkage-corrected coordinates instead (red). *I*_exp_(*q*) is also shown for comparison. Calculating *I*_img_(*q*) using the shrinkage-corrected coordinates results in a far better agreement with *I*_exp_(*q*) at low *q*.

Another apparent discrepancy between *I*_img_(*q*) and *I*_exp_(*q*) in this case study is the misaligned structure-factor peak in the low-*q* region at around 0.01 Å^−1^. In *I*_img_(*q*), the structure-factor peak appears at *q* ≈ 0.013, compared to *q* ≈ 0.01 in *I*_exp_(*q*). We believe that this discrepancy can be attributed to shrinkage of the cornea-slice sample, which likely occurred during its preparation for imaging by EM. In Fig. 4, we show that multiplying the coordinates obtained from the EM image by a scale factor of 1.225, and then recalculating *g*(*r*) and *S*(*q*) using these scaled coordinates results in a much better match between the experimental and image-obtained structure-factor peak. It is likely that shrinkage of the matrix surrounding the fibrils was the primary cause of this contraction, since the form-factor peaks in *I*_img_(*q*) remain unchanged by the scaling of the EM-generated coordinates, and fibril diameters obtained from EM and SAS are in good agreement despite shrinkage. We refer the reader to the ESI† for an animation that illustrates how the structure-factor peak in *I*_img_(*q*) moves to the correct position as we slowly increase the coordinate scaling factor from 1.0 to 1.225. The fact that EM images may not represent the native states (absent of any chemical modification) of the samples being imaged highlights a limitation of our method, where it may not always be possible to obtain accurate SAS intensities owing to morphological changes which occur during EM-based sample-preparation procedures.

## 3 Applications, limitations and conclusion

The ability to quickly characterize a sample for which one already has EM data can be a clear boon for the design and implementation of SAS experiments. The proposed method could allow the feasibility of future experimental SAS measurements to be evaluated – and would inform the conduct of time and resource-effective SAS measurements, by elucidating, for example, the required q-range, which will dictate the wavelength and sample-to-detector distances used during the experiment. It could also be used to indicate how such datasets may be analyzed, aiding in model selection and elucidating the contributions of different components in more complex samples.

We discussed some factors that should be taken into consideration when applying the methods outlined in this work, *i.e.*, particle sizes measured by EM can be unreliable and sample-preparation procedures for EM can cause morphological changes which result in inaccurate image-obtained SAS intensities. Additional factors that may not be obvious from the methodology or case studies are as follows. In order for our method to be applied, a sufficient number of particles is necessary to calculate *g*(*r*) and hence *S*(*q*). We found that a minimum of around 200 or more particles should be present in an image to ensure that one can obtain a smooth and noise-free radial-distribution function. This number is a lower bound and the quality of *g*(*r*) improves further as this number is increased. However, there may be situations where the structure factor may be disregarded owing to scatterers being arranged at complete random in the sample. In this case, *S*(*q*) = 1 at all values of *q*, and the form factor may be computed from a very small number of particles in the image. An additional limitation is that our method can not be applied if the area being imaged is a 2-D cross-section of a 3-D sample (barring the case of parallel and infinitely long cylinders) where the scatterers protrude into the 2-D image plane. For example, suppose that a sample, which contains monodisperse spherical nanoparticles distributed in a matrix, was sliced along a plane for imaging. The spherical particles that intersected the plane were likely at different distances from it, resulting in the cross-sections of each sphere appearing as circles of different sizes. It would therefore not be possible to infer the true sizes of the nanoparticles from an electron micrograph and calculate the correct form factor required to calculate *I*(*q*). Besides, the 2-D slice in this case provides no information about the spatial distributions of the nanoparticles below the image plane, and hence the true structure factor also can not be inferred. A future prospect of this work involves extending it to 3-D EM images such as those obtained by tomography or focused ion-beam scanning electron microscopy. Unlike 2-D particle segmentations, their 3-D counterparts contain all the information necessary to determine the morphological parameters required in the calculation of the form factor. Furthermore, the extension to 3-D enables us to move beyond the two specific cases studied in this paper, and calculate SAS intensities of any sample so long as it can be segmented into regions from which we can calculate *P*(*q*) and *S*(*q*). Consequently, samples that are more generic could be modeled while making fewer assumptions. Nonetheless, this paper showcases an important step towards this ultimate goal and lays the core foundations for this future work. We provide a Python notebook of the implementation which outlines the steps taken to go from image to SAS intensity at https://github.com/by256/i2sas.

## Conflicts of interest

There are no conflicts to declare.

## Supplementary Material

RA-012-D2RA00685E-s001
